# The sensitive and selective electrochemical detection of carcinoembryonic antigen using a nanoMIPs-aptamer sandwich assay

**DOI:** 10.1038/s41598-025-22971-7

**Published:** 2025-11-10

**Authors:** Chutimon Akkapinyo, Yingyot Poo-arporn, Ramida Rattanakam, Kittitat Subannajui, Peter A. Lieberzeit, Peter Wolschann, Rungtiva P. Poo-arporn

**Affiliations:** 1 Biological Engineering Program, Faculty of Engineering, King Mongkut’s University of Technology Thonburi, Bangkok, 10140 Thailand; 2 Synchrotron Light Research Institute (Public Organization), Nakhon Ratchasima, 30000 Thailand; 3 Department of Chemistry, Faculty of Science, Kasetsart University, Bangkok, 10900 Thailand; 4 School of Materials Science and Innovation, Faculty of Science, Mahidol University, Bangkok, 10400 Thailand; 5 Department of Physical Chemistry, Faculty for Chemistry, University of Vienna, 1090 Vienna, Austria; 6 Institute of Theoretical Chemistry, University of Vienna, Vienna, 1090 Austria

**Keywords:** Cancer, Carcinoembryonic antigen, Metal organic framework, Molecularly imprinted polymer, NanoMIPs, Biochemistry, Biomarkers, Biotechnology, Cancer, Chemistry, Materials science, Nanoscience and technology

## Abstract

Carcinoembryonic antigen (CEA) is a well-established cancer biomarker that plays a crucial role in cancer diagnosis, treatment monitoring, and recurrence detection. This study presents the development of a highly sensitive and selective electrochemical sensor based on molecularly imprinted polymer nanoparticles (nanoMIPs) for CEA detection. CEA-specific nanoMIPs were synthesized and immobilized onto a screen-printed carbon electrode, providing selective recognition sites for CEA binding. The electrochemical signal probe was constructed using a metal organic framework, UiO-66-NH_2_, which served as the substrate for lead ion (Pb^2+^) adsorption and aptamer functionalization. A nanoMIPs-aptamer sandwich assay was used for CEA detection. Square wave anodic stripping voltammetry was used to measure the electrochemical response of Pb^2+^, which correlates with the amount of CEA captured on the electrode surface. The sensor demonstrated an excellent linear CEA detection range at concentrations between 1 and 1,000 ng/mL. The limit of detection was determined to be 1.4 ng/mL, which is below the clinical cut-off value for CEA. The proposed sandwich assay offers several advantages, including low cost, high sensitivity, good reproducibility, and excellent selectivity. When applied to CEA-spiked human serum samples with the appropriate pretreatment, the sensor achieved satisfactory recovery rates ranging from 98.12 to 103.24%, highlighting its applicability for clinical diagnostics.

## Introduction

Cancer is a leading cause of morbidity and mortality worldwide, accounting for nearly 10 million deaths according to the latest data from the World Health Organization (WHO)^1^. Furthermore, the estimated number of new cancer cases is projected to increase by up to 55% between 2022 and 2045^2^. Consequently, the effective diagnosis and treatment of this disease are crucial for reducing patient mortality. The detection of cancer biomarkers plays an extremely important role in early diagnosis, disease monitoring, and recurrence detection. Carcinoembryonic antigen (CEA) is one of the most commonly used biomarkers for cancer detection^3^. CEA is a glycoprotein with a molecular weight of 180–200 kDa; it is produced during fetal development, but its expression is largely inhibited after birth^3,4^. The abnormal expression of CEA is associated with a variety of malignancies, including gastric, breast, ovarian, lung, pancreatic, and especially colorectal cancer^5,6^. The CEA level in healthy people is typically less than 5 ng/mL^7^, while cancer patients tend to exhibit CEA levels that exceed 20 ng/mL^5^. In addition, elevated levels of CEA in serum are associated with tumor progression and are commonly used for prognosis monitoring and treatment evaluation^6,8^. Therefore, the development of sensing platforms that are capable of rapid and accurate CEA detection is extremely promising for improving cancer diagnostics.

Conventional methods for CEA detection are based on immunoassay techniques such as the enzyme-linked immunosorbent assay (ELISA)^9^, the radioimmunoassay (RIA)^10^, and the fluorescence immunoassay^11^. Although these techniques are well-established, widely accepted, and highly selective, they are still limited by their high detection costs and the need for trained professionals to conduct these assays. In addition, some immunoassays require labeling molecules, such as fluorescent tags, radioactive elements, or enzymes that can generate signals that correspond to specific immune reactions. These labeling agents often require complex and costly instrumentation for signal analysis, resulting in complicated procedures. Furthermore, the use of radioactive elements can have adverse health effects^6^. Recent technological advancements in electrochemical methods have shown that they are promising alternatives, offering advantages such as their rapid response, ease of use, low cost, and high sensitivity. Consequently, electrochemical sensors based on immunoassays have been proposed for CEA detection^12–16^. However, because antibodies are required for sensor selectivity, they are subject to inherent limitations such as high costs and complex handling, which pose challenges for certain applications. Thus, the development of sensors with rapid response, high sensitivity, high selectivity, and low cost is crucial for effective CEA detection in cancer surveillance and disease monitoring.

Molecularly imprinted polymers (MIPs) are synthetic polymers that are engineered to bind specifically to their target molecules. These imprinted sites are complementary to their target analytes in terms of shape, size, and functional group interactions^17^. MIPs offer several advantages over antibodies, including low production costs, ease of synthesis, and stability at room temperature, allowing for mass production^18,19^. These advantageous properties have led to the use of MIPs as recognition elements in place of antibodies to develop biosensors for various diseases, including cardiovascular diseases^20^, Alzheimer’s disease^21^, and breast cancer^22^. Electrochemical MIP-based sensors have also been proposed for the detection of CEA. Electropolymerization techniques have been employed to fabricate MIP films on electrode surfaces using various monomers, including pyrrole^23^, aminophenol^24^, gallic acid^25^, dopamine^26,27^, and o-phenylenediamine^28,29^. Although electropolymerization allows for MIP synthesis under mild conditions, each electrode must be fabricated individually, making it unsuitable for mass production. Furthermore, these are quality control challenges due to the potential variation between sensors.

In addition to thin film formats, MIPs designed for CEA detection have also been developed in the form of core-shell nanoparticles. For example, Fe_3_O_4_ nanoparticles^30^, copper metal-organic frameworks (Cu-MOF)^31^, and silver nanoparticle-deposited silica nanospheres (SiO_2_@AgNPs)^32^ have all been employed as cores for MIP coatings. Core-shell nanoparticle MIPs can be more easily mass-produced, and their applications are not limited to electrode surfaces: these particles can be integrated with other types of transducers. Furthermore, solid-phase synthesis allows for the production of molecularly imprinted polymer nanoparticles (nanoMIPs) without the need for core particles. Due to their nanoscale size, nanoMIPs exhibit several outstanding properties, including high surface area-to-volume ratios, the facile elution of template proteins, rapid binding kinetics, excellent dispersibility, and high compatibility with various nanodevices^33–35^. In addition, nanoMIPs can be produced on a large scale in a single batch, addressing the issues related to sensor production time and stability. Notably, nanoMIPs have not yet been applied to CEA detection; the synthesis of nanoMIPs with high specificity toward CEA represents a novel challenge in this field. This study aims to fill this research gap by developing an electrochemical MIP-based sensor that utilizes nanoMIPs for the detection of CEA.

Metal organic frameworks (MOFs) are crystalline porous materials composed of metal ions or clusters that are coordinated to organic linkers, forming three-dimensional networks. MOFs have been extensively employed in the fabrication of sensors for CEA detection due to their large specific surface area, excellent chemical stability and tunability, high porosity, ease of surface modification, and adjustable size and shape^7^. Indeed, previous studies have modified electrode surfaces with MOFs to immobilize recognition molecules such as antibodies^36,37^ or aptamers^38,39^, thereby creating specific binding sites for CEA. In addition, the high porosity of MOFs allows for the efficient loading of labeling agents. MOFs can also serve as substrates for signal probe preparation through functionalization of antibodies^40,41^ or aptamers^42,43^. In particular, UiO-66-NH_2_-type MOFs contain an abundance of amine functional groups derived from their organic linker, 2-aminoterephthalic acid, allowing for excellent chelation with metal ions^44,45^. In addition, the zirconium nodes in UiO-66-NH_2_ facilitate aptamer functionalization via Zr-O-P bonding^46,47^. Furthermore, UiO-66-NH_2_ can be readily synthesized in the laboratory. These outstanding properties make UiO-66-NH_2_ a promising substrate for signal probe preparation. Although the amine functional groups of UiO-66-NH_2_ allow chelation with several metal ions, preferential adsorption of Pb^2+^ has been reported. Several experimental reports show higher adsorption capacities^48^ and distribution coefficients^49^ for Pb^2+^ compared with other ions (e.g., Cd^2+^, Cr^3+^, Hg^2+^). Moreover, density functional theory (DFT) calculations revealed a lower adsorption energy of Zr-based MOFs toward Pb^2+^ than for other heavy metals (Cu^2+^, Zn^2+^, Cd^2+^ and Hg^2+^)^50^. Therefore, Pb^2+^ is an ideal choice to serve as the signal probe in the electrochemical detection system.

In this study, a nanoMIPs-aptamer sandwich assay was developed for the detection of CEA. Specifically, nanoMIPs were synthesized to serve as CEA-specific recognition sites and immobilized onto a screen-printed carbon electrode (SPCE) to fabricate an electrochemical MIP-based sensor. SPCEs offer several advantages, including low fabrication cost, ease of operation, disposability, and practical convenience. Electrochemical detection was based on the signal generated by lead ions loaded in the signal probe. This signal can be measured once the nanoMIPs-aptamer sandwich complex has been formed, and corresponds to the concentration of the target CEA captured on the electrode surface. In addition, the use of the signal probe improves the accuracy of the developed sensor for CEA quantification.

## Materials and methods

### Materials

CEA was purchased from Medix Biochemica. A 5′-phosphate-modified CEA-specific aptamer with the sequence 5′-Phos-C6-ATACCAGCTTATTCAATT-3′ was obtained from Integrated DNA Technologies, Inc. (IDT). Carboxylic acid-functionalized multi-walled carbon nanotubes (MWCNTs), lead (II) nitrate, zirconium (IV) chloride, 2-aminoterephthalic acid, silica gel (70–230 mesh), (3-aminopropyl)triethoxysilane (APTES), acrylic acid, N-isopropylacrylamide (NIPAm), *N*-tert-butylacrylamide (TBAm), *N*,* N′*-methylenebisacrylamide (BIS), *N*,* N*,*N′N′*-tetramethylethylenediamine (TEMED), *N*-(3-dimethylaminopropyl)-*N′*-ethylcarbodiimide (EDC), dimethyl sulfoxide (DMSO), dimethylformamide (DMF) and glutaraldehyde were purchased from Sigma Aldrich. Ammonium persulfate (APS) was obtained from Acros Organics. A standard CEA solution was prepared in 0.01 M phosphate buffer saline (PBS; pH 7.4). An SPCE with a three-electrode configuration was purchased from Quasence Co., Ltd. (Bangkok, Thailand).

### Apparatus

All electrochemical measurements were performed using an AutoLab PGSTAT128N system (EcoChemie B.V., Utrecht, Netherlands). Scanning electron microscopy (SEM) and energy-dispersive X-ray spectroscopy (EDX) were conducted on a Phenom Pharos Desktop FEG-SEM (ThermoFisher Scientific, Netherlands). Dynamic light scattering (DLS) and zeta potential measurements were carried out using a nanoparticle analyzer (SZ-100V2, HORIBA Scientific). Fourier transform infrared spectroscopy (FTIR) was performed using a Thermo Scientific Nicolet iS5 (Thermo Fisher Scientific, Waltham, MA, USA).

### Synthesis of nanomips

#### Immobilization of CEA on silica gels

CEA-specific nanoMIPs were synthesized using the solid-phase synthesis method proposed by Canfarotta et al.^51^, with slight modifications. The hydroxyl groups on the surface of silica gel were activated by treatment with NaOH. Briefly, 10 g of silica gel was mixed with 20 mL of 1 M NaOH and heated at 105 ℃ for 20 min. The activated silica gels were transferred to a filter funnel and washed sequentially with 200 mL of deionized (DI) water, 200 mL of PBS, 600 mL of DI water, and 400 mL of acetone. The particles were dried on a filter funnel connected to a vacuum source. 2 g of the activated silica gel was then transferred to a new tube and incubated in 4 mL of 2.5% (v/v) APTES solution in anhydrous toluene for 24 h at room temperature (RT). Functional amino groups were introduced onto the surface of silica gel via APTES modification. Following this, the silanized silica gel was washed sequentially with 80 mL of acetone and 20 mL of an ethanol/acetone (1:1, v/v) mixture. The particles were subsequently incubated in 4 mL of 7.5% glutaraldehyde solution prepared in 0.01 M PBS (pH 7.4) at RT for 2 h. During this step, the particles turned orange, indicating that the functionalization of aldehyde groups on the surface was successful. The unreacted glutaraldehyde was removed by rinsing the solution with 100 mL of PBS. The activated silica gel was then incubated overnight at 4 °C in 4 mL of CEA solution (0.1 mg/mL) prepared in 10 mM PBS (pH 7.4). The template CEA was immobilized onto the surface of the activated silica gel through the reaction between the aldehyde groups on the silica surface and the primary amine groups of the protein template. The excess CEA template was removed by washing with 100 mL of PBS, followed by 100 mL of DI water. The unreacted aldehyde groups on the silica surface were blocked by incubating the gels with 4 mL of 50 mM ethanolamine solution for 30 min. The excess ethanolamine was removed by rinsing with 100 mL of DI water. Finally, the CEA-functionalized silica gels were stored at −20 °C until further use.

#### Polymerization of nanomips

**Fig. 1:**
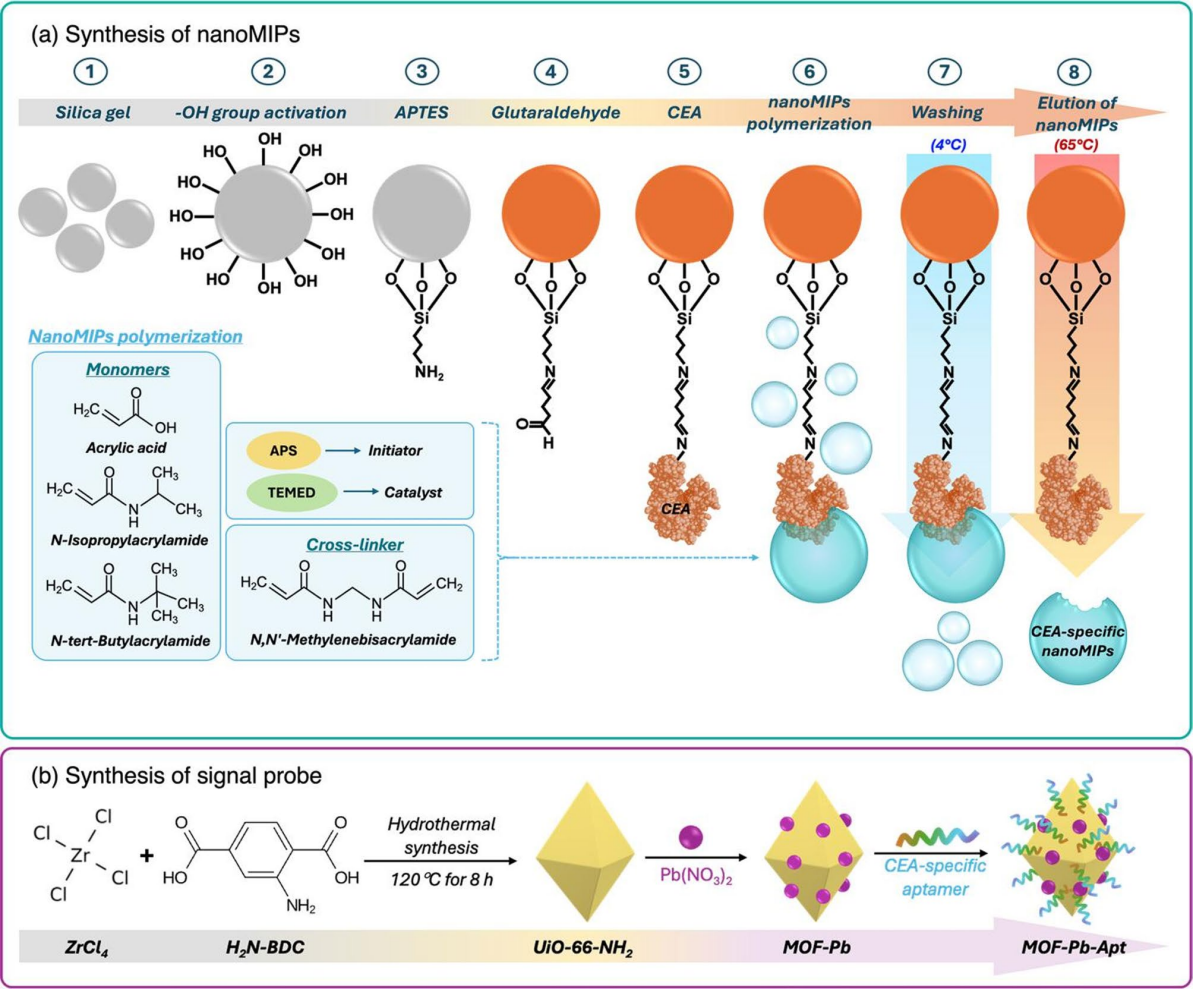
Schematic representation of (**a**) the solid-phase synthesis of nanoMIPs and (**b**) the preparation of the signal probe, MOF-Pb-Apt.

The polymer mixture was prepared by combining 15.1 µL of acrylic acid, 100 µL of NIPAm (500 mg/mL in DMSO), 300 µL of TBAm (143.3 mg/mL in DMSO), 100 µL of BIS (31.0 mg/mL in DMSO), and 2,484.9 µL of Milli-Q water. The resulting solution was sonicated until all components were completely dissolved. Following this, 45 µL of APS (200 mg/mL) and 45 µL of 20% (v/v) TEMED were added to the solution. The CEA-immobilized silica gel was added after mixing. The mixture was purged with N₂ gas for 10 min and then placed on a rotator at RT for 2 h to complete the polymerization. The mixture was then transferred into a solid-phase extraction (SPE) cartridge equipped with a 100 μm polyethylene (PE) frit for subsequent elution. Unreacted chemicals and non-imprinted nanoparticles were removed by washing with 50 mL of cold Milli-Q water (4 °C). Subsequently, 50 mL of Milli-Q water at 65 °C was used to elute the high-affinity CEA-specific nanoMIPs. Non-imprinted polymer nanoparticles (nanoNIPs) were synthesized using the same method but without the steps involving glutaraldehyde and CEA. A schematic representation of the nanoMIP synthesis process is shown in Fig. 1a.

### Synthesis of the signal probe MOF-Pb-Apt

#### Synthesis of UiO-66-NH_2_

First, 43 mg of H_2_N-BDC and 15 mg of ZrCl_4_ were dissolved in 4 mL of DMF at RT. The resulting solution was sonicated for 10 min before being transferred into a 10 mL Teflon-lined stainless steel autoclave, after which 300 µL of acetic acid was added. The solution was heated at 120 °C for 8 h and then cooled to RT. The synthesized nanoparticles were filtered and washed three times with 10 mL of ethanol and deionized (DI) water. The resultant yellow powder was dried under vacuum at RT.

#### Adsorption of Pb^2+^ and functionalization of CEA-specific aptamer onto UiO-66-NH_2_

A 2 mg sample of the synthesized UiO-66-NH_2_ was dispersed in 1 mL of DI water. Following this, 1 mL of 30 mM Pb(NO_3_)_2_ was added, and the mixture was stirred at 200 rpm overnight. The suspension was then centrifuged at 13,000 rpm for 10 min to collect the Pb^2+^-adsorbed UiO-66-NH_2_ (MOF-Pb). The MOF-Pb was then resuspended in 1 mL of 10 mM Tris-HCl buffer (pH 7.4). Following this, 1 mL of 500 nM 5′-phosphate-modified CEA-specific aptamer (prepared in the same buffer) was introduced, after which the mixture was incubated with shaking at 200 rpm for 8 h. Unbound aptamer was removed by centrifugation at 10,000 rpm for 5 min, and the pellet was washed twice with 10 mM Tris-HCl buffer (pH 7.4) containing 0.1 M NaCl. Finally, the particles were washed once more with 10 mM Tris-HCl buffer without NaCl and redispersed in 2 mL of Tris-HCl buffer. The prepared signal probe (MOF-Pb-Apt) was kept at 4 °C until further use. A schematic representation of MOF-Pb-Apt preparation is demonstrated in Fig. 1b.

### Preparation of nanoMIPs/APTES/OH/MWCNTs/SPCE

**Fig. 2:**
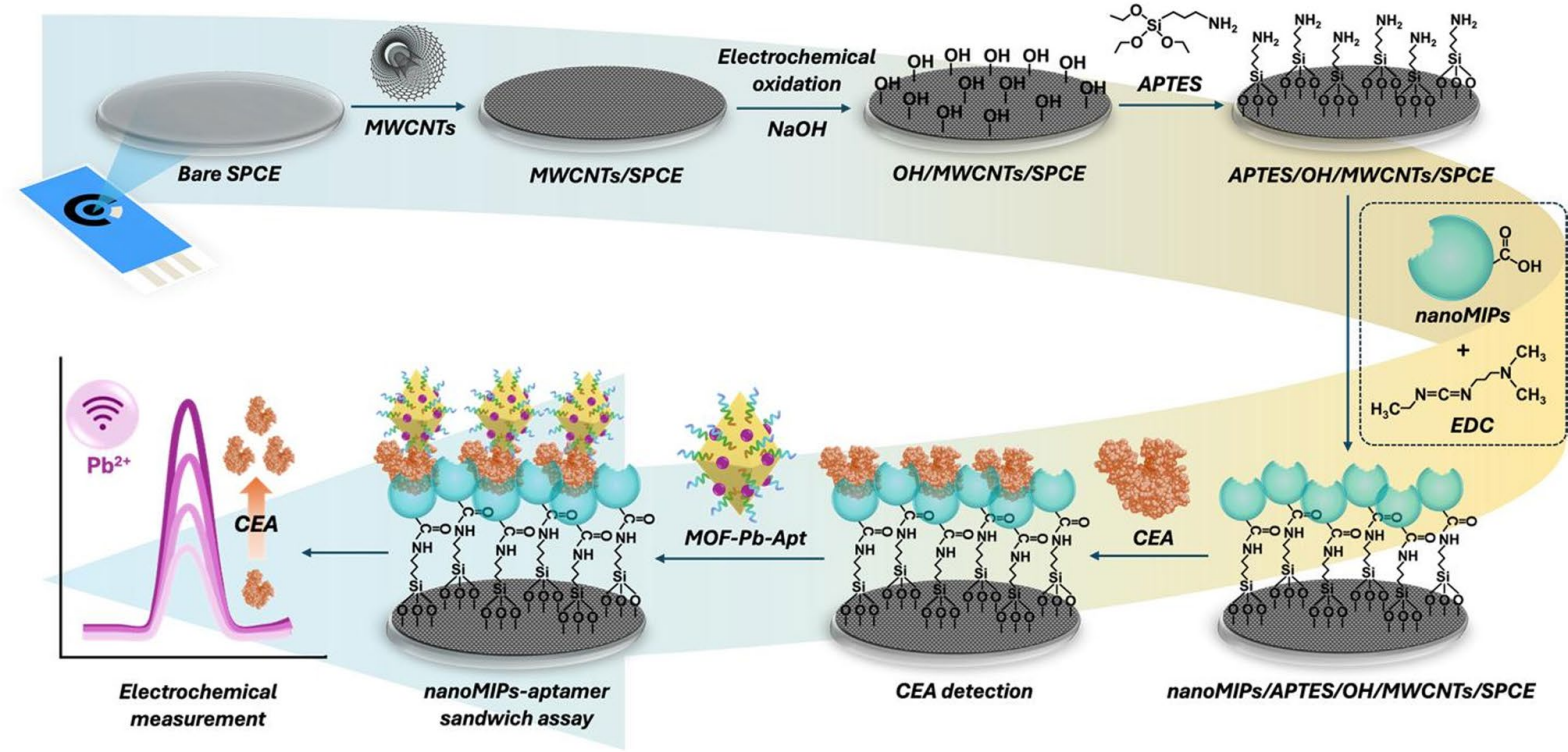
Schematic representation of the sensor fabrication process and the CEA detection strategy utilized by the nanoMIPs-aptamer sandwich assay.

The fabrication process of the nanoMIPs/APTES/OH/MWCNTs/SPCE sensor is presented in Fig. 2. A 3 µL aliquot of 0.1 mg/mL MWCNTs prepared in 40% (v/v) ethanol was dropped onto the surface of the SPCE, and the modified electrode was dried at RT. Electrochemical oxidation was performed in 0.1 M NaOH by applying a potential from 0.1 to 0.7 V for 30 cycles at a scan rate of 100 mV/s using the cyclic voltammetry (CV) technique; this process activates the hydroxyl groups on the MWCNTs/SPCE surface. The resulting OH/MWCNTs/SPCE was rinsed with DI water and dried under a stream of N_2_ gas. Amine functional groups were then introduced by incubating the electrode with 5 µL of 3% (v/v) APTES for 1 h. Excess APTES was removed by rinsing the modified electrode (APTES/OH/MWCNTs/SPCE) thoroughly with DI water. The synthesized CEA-specific nanoMIPs were immobilized onto the APTES/NaOH/MWCNTs/SPCE electrode via amide bond formation. Specifically, 200 µL of the CEA-specific nanoMIPs (0.6 mg/mL) was mixed with 20 µL of EDC (25 mg/mL) and applied to the APTES/NaOH/MWCNTs/SPCE. The electrode was incubated for 3 h to allow for covalent coupling. Excess nanoMIPs were removed by rinsing with DI water. The resulting nanoMIPs/APTES/OH/MWCNTs/SPCE electrode was dried under a stream of N_2_ and stored under dry conditions. The nanoNIPs-modified electrode was prepared by following the same immobilization procedure but using nanoNIPs rather than nanoMIPs.

### NanoMIPs-aptamer sandwich assay procedure

CEA detection was performed by incubating 10 µL of CEA sample on the prepared nanoMIPs/APTES/OH/MWCNTs/SPCE for 1 h. The unbound CEA was then removed by washing with PBS containing 0.05%v/v TWEEN20 for 5 min, followed by rinsing with DI water. Subsequently, 5 µL of the synthesized MOF-Pb-Apt signal probe was added to perform the nanoMIPs-aptamer sandwich assay, followed by incubation for 30 min. The excess signal probe was removed by rinsing with DI water. The electrochemical measurement was carried out using square wave anodic stripping voltammetry (SWASV) in an acetate buffer (pH 4.5). Deposition was performed at a potential of −1.2 V for 120 s, followed by a stripping step, which was recorded from − 1.2 V to 0.0 V with a step potential of 0.004 V, an amplitude of 0.025 V, and a frequency of 25 Hz.

### Real sample analysis

Commercial human serum was used to evaluate the sensor’s performance in the context of clinical detection. Samples were prepared by spiking CEA into commercial human serum at concentrations of 5, 10, and 25 ng/mL. To minimize interference from high concentrations of human serum albumin (HSA), the spiked samples were treated using a 100 kDa molecular weight cut-off filter. The treated samples were then adjusted to their original volume with PBS (pH 7.4), and 10 µL of each sample was applied to the developed sensor for testing.

## Results and discussions

### Characterization of the synthesized nanomips

**Fig. 3:**
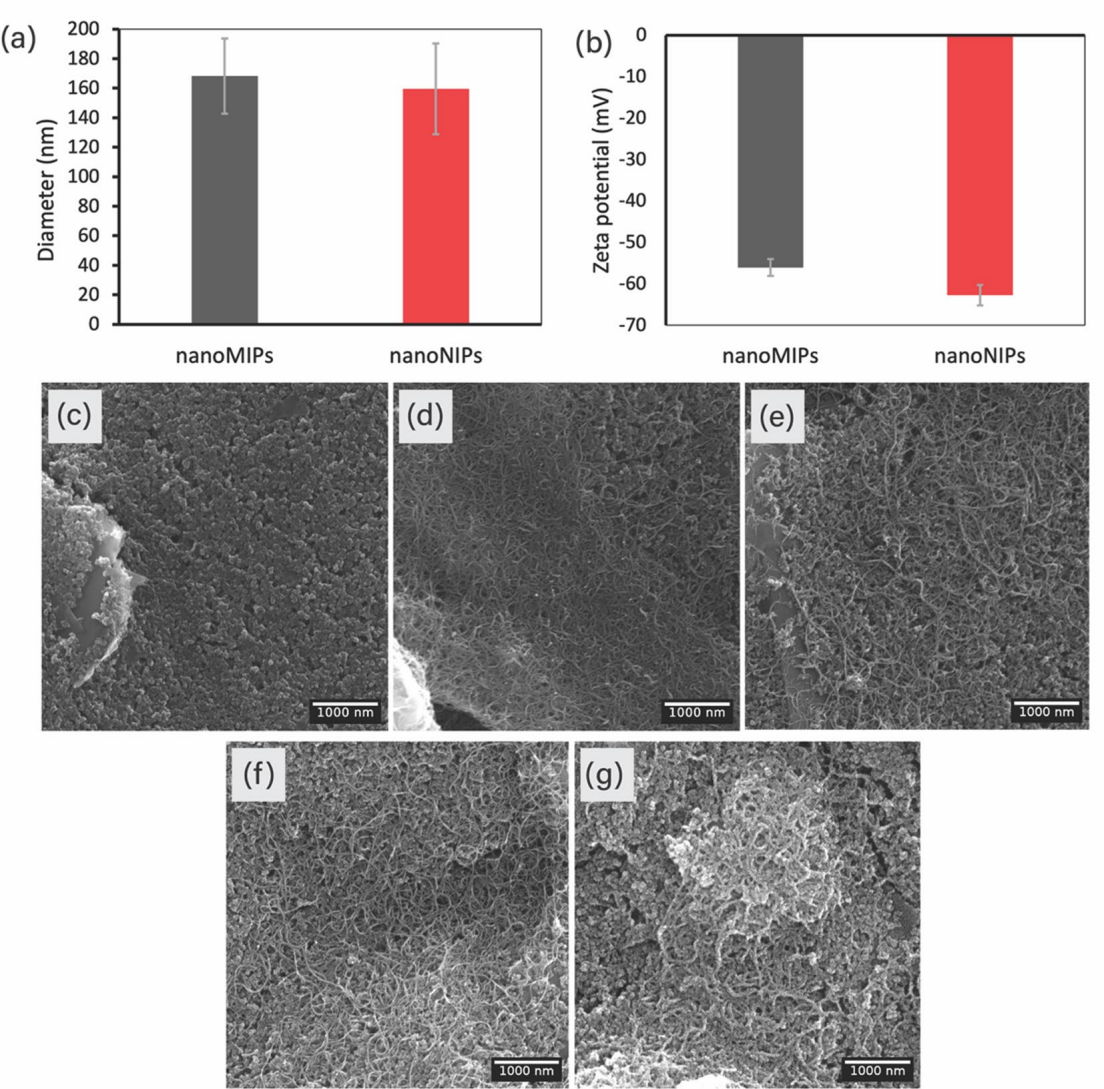
(**a**) DLS and (**b**) zeta potential measurements of the synthesized nanoMIPs and nanoNIPs. SEM images of (**c**) bare SPCE, (**d**) MWCNTs/SPCE, (**e**) OH/MWCNTs/SPCE, (**f**) APTES/OH/MWCNTs/SPCE, and (**g**) nanoMIPs/APTES/OH/MWCNTs/SPCE.

The synthesized CEA-specific nanoMIPs and their corresponding non-imprinted control particles (nanoNIPs) were characterized by DLS and zeta potential measurements as demonstrated in Fig. 3a,b, respectively. The hydrodynamic diameters of the nanoMIPs and nanoNIPs were 168.2 ± 25.5 nm and 159.5 ± 30.8 nm, respectively. The slightly larger size of the nanoMIPs was attributed to the molecular imprinting process. The polydispersity index (PDI) values of the nanoMIPs and nanoNIPs were 0.686 and 0.635, respectively, indicating moderate particle dispersity. The surface charge and colloidal stability of the synthesized nanoMIPs and nanoNIPs were evaluated by zeta potential measurement; these were found to be −56.1 ± 2.0 mV and − 62.8 ± 2.5 mV for the nanoMIPs and nanoNIPs, respectively. Negative zeta potential measurements represent the negative charge of the carboxyl groups present in the acrylic acid monomer^52^. The slightly more negative zeta potential observed in the nanoNIPs may be due to structural differences resulting from the absence of the molecular imprinting process. Finally, high negative zeta potential values are indicative of strong electrostatic repulsion between particles, contributing to their colloidal stability in solution.

### Morphology characterization of electrode modification

The surface morphology of the modified electrodes was analyzed using SEM. SEM images of bare SPCE, MWCNTs/SPCE, OH/MWCNTs/SPCE, APTES/OH/MWCNTs/SPCE, and nanoMIPs/APTES/OH/MWCNTs/SPCE are presented in Fig. 3c,g, respectively. The bare SPCE exhibited a rough surface composed of graphite flakes and the binding agent from the carbon ink. Following MWCNT deposition, the surface of the MWCNTs/SPCE displayed a spaghetti-like morphology characteristic of MWCNTs, indicating that they had been successfully physically adsorbed onto the SPCE surface. No obvious morphological differences were observed in the OH/MWCNTs/SPCE and APTES/OH/MWCNTs/SPCE compared to the MWCNTs/SPCE. However, there was a notable increase in surface roughness observed in the nanoMIPs/APTES/OH/MWCNTs/SPCE, which appeared to coat the spaghetti-like structure of the MWCNTs. These results confirm the successful stepwise modification of the SPCE surface with MWCNTs and nanoMIPs.

### Electrochemical characterization of electrode modification

**Fig. 4:**
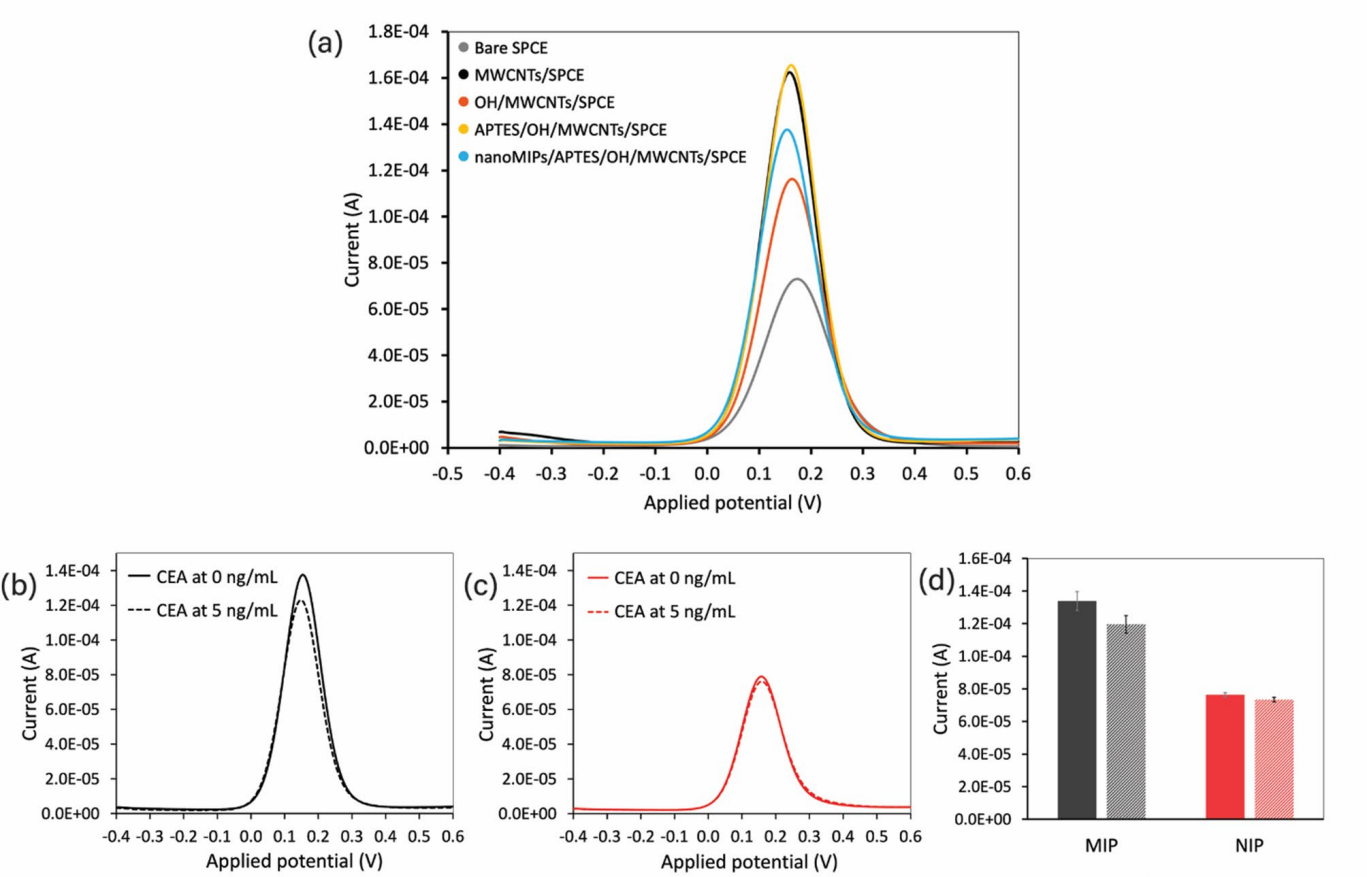
(**a**) Comparison of 5 mM [Fe(CN)_6_]^3−/4−^ SWV voltammograms obtained at each of the SPCE modification steps: bare SPCE, MWCNTs/SPCE, OH/MWCNTs/SPCE, APTES/OH/MWCNTs/SPCE, and nanoMIPs/APTES/OH/MWCNTs/SPCE. Comparison of SWV voltammograms of (**b**) nanoMIPs/APTES/OH/MWCNTs/SPCE and (**c**) nanoNIPs/APTES/OH/MWCNTs/SPCE after rebinding with CEA at concentrations of 0 and 5 ng/mL. (**d**) A comparison of the corresponding current responses.

To characterize the electrochemical behavior of the modified electrodes, square wave voltammetry (SWV) was performed using a 2.5 mM [Fe(CN)_6_]^3−/4−^ solution as the redox probe. The potential was scanned from − 0.4 to 0.7 V using an amplitude of 0.05 V, a step potential of 0.005 V, and a frequency of 5 Hz. All stepwise modifications, including bare SPCE, MWCNTs/SPCE, OH/MWCNTs/SPCE, APTES/OH/MWCNTs/SPCE, and nanoMIPs/APTES/OH/MWCNTs/SPCE, were electrochemically characterized; the results of these analyses are presented in Fig. 4a. All electrodes exhibited well-defined oxidation peaks of the redox probe; however, the current responses varied depending on their surface modifications. For example, the MWCNTs/SPCE exhibited a higher current response compared to the bare SPCE due to the high surface area and excellent conductivity of MWCNTs, which enhances electron transfer at the electrode surface. In contrast, the current response decreased after electrochemical oxidation in NaOH due to the negatively charged hydroxyl groups introduced during the activation step, which repelled the redox probe and hindered electron transfer on the OH/MWCNTs/SPCE. The current response increased following the successful preparation of APTES/OH/MWCNTs/SPCE and the enhancement of electron transfer efficiency^53^. This phenomenon can be explained by the favorable electrostatic interaction between the positively charged protonated amino groups of APTES and the negatively charged anionic probe [Fe(CN)_6_]^3−/4−^^54,55^. The current response decreased once again following the subsequent immobilization of nanoMIPs due to their polymeric structure, which impedes electron transfer at the electrode surface, resulting in lower current responses for the nanoMIPs/APTES/OH/MWCNTs/SPCE. The SWV results confirmed the successful assembly of MWCNTs, hydroxyl groups, APTES, and nanoMIPs on the electrode surface.

### Performance of the synthesized nanomips for CEA detection

Following the successful modification of nanoMIPs on the electrode surface, their CEA detection capabilities were evaluated using SWV with a 2.5 mM [Fe(CN)_6_]^3−/4−^ redox probe. The prepared nanoMIPs/APTES/OH/MWCNTs/SPCE and nanoNIPs/APTES/OH/MWCNTs/SPCE were tested with a CEA concentration of 5 ng/mL and compared to the background signal (i.e., 0 ng/mL CEA). The electrochemical results are presented in Fig. 4b and d. There was a clear decrease in current response observed in the nanoMIPs/APTES/OH/MWCNTs/SPCE upon exposure to 5 ng/mL CEA, while only a slight decrease was observed in the nanoNIPs/APTES/OH/MWCNTs/SPCE. The current change observed in the nanoMIPs-modified electrode was 14.22 µA—this was approximately 4.8 times greater than the values obtained from the nanoNIPs-modified electrode (2.95 µA). This higher current change highlights the superior sensitivity of nanoMIPs in capturing the target CEA molecules. Furthermore, these results confirm the specific binding capabilities and sensing performance of the nanoMIPs-based electrochemical sensor for CEA detection developed in this study.

### Characterization of the signal probe

**Fig. 5:**
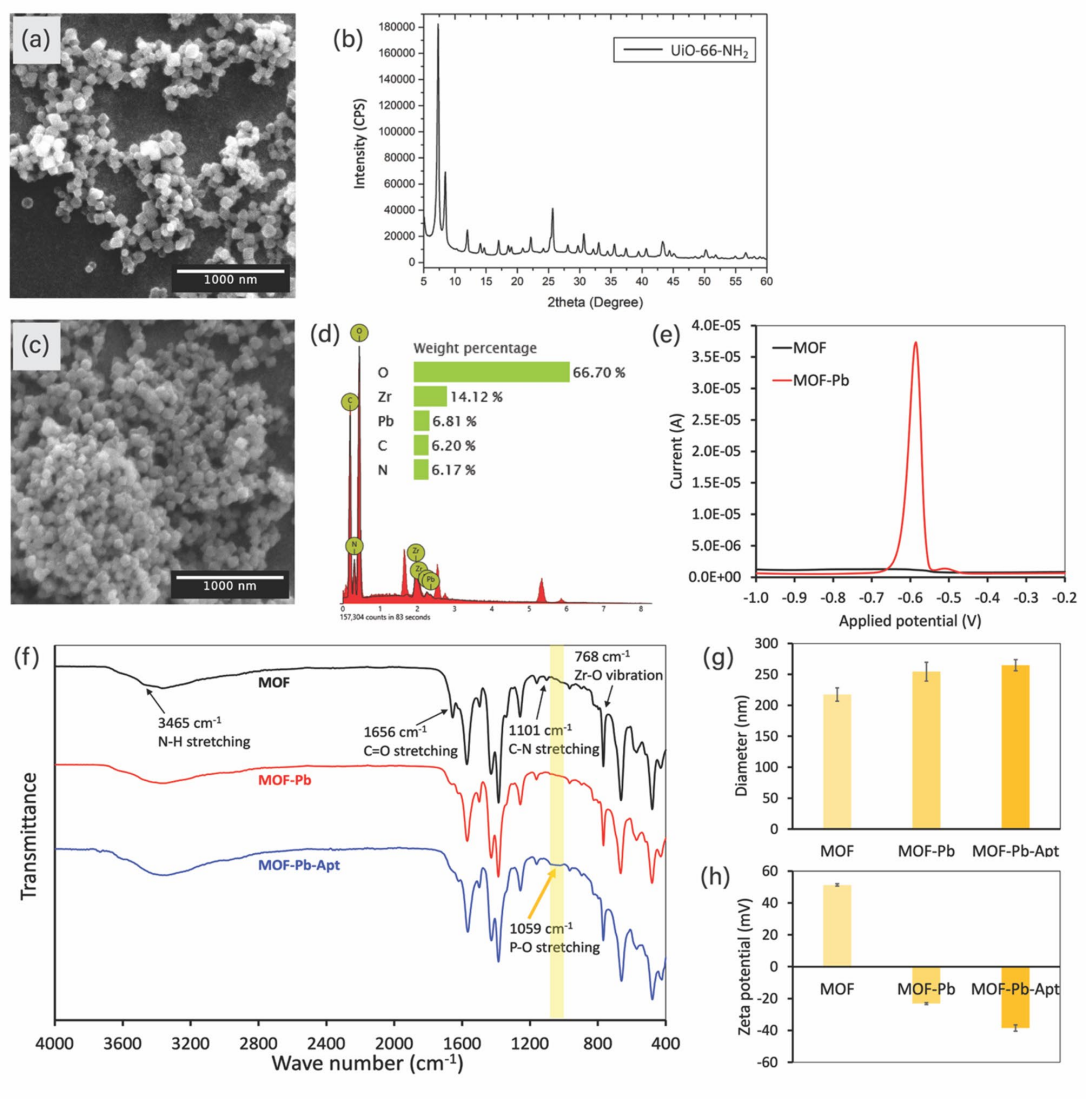
(**a**) SEM images and (**b**) XRD spectrum of UiO-66-NH_2_. (**c**) SEM image and (**d**) EDS analysis of MOF-Pb, and (**e**) comparison of SWASV voltammograms obtained from MOF and MOF-Pb. (**f**) Comparison of FTIR spectra, (**g**) hydrodynamic diameters, and (**h**) zeta potential measurements of MOF, MOF-Pb, and MOF-Pb-Apt.

The surface morphology of the synthesized UiO-66-NH_2_ was characterized using SEM (Fig. 5a). The SEM images revealed that the surface of the UiO-66-NH_2_ exhibited an octahedral-like structure with uniform size and a smooth surface. Figure 5b presents the X-ray Diffraction (XRD) pattern of the synthesized UiO-66-NH_2_. Characteristic diffraction peaks were observed at 7.34° and 8.48°, which are consistent with the diffraction peaks of UiO-66-NH_2_. The sharp and narrow nature of the peaks indicates a high degree of crystallinity^44,45^. Furthermore, the surface area and pore diameter of the synthesized UiO-66-NH_2_ were evaluated using Brunauer-Emmett-Teller (BET) and Barrett-Joyner-Halenda (BJH) methods, respectively (data not shown). The surface area was calculated to be 984.218 m^2^/g based on the BET equation, while BJH analysis suggested an average pore diameter of 2.968 nm. The obtained surface area and pore diameter were in good agreement with previously reported data for UiO-66-NH_2_^44,56^. The SEM, XRD, BET, and BJH results indicate the successful synthesis of UiO-66-NH_2_ with uniform particle morphology, high crystallinity, large surface area, and a nanoporous structure.

The adsorption of Pb^2+^ onto UiO-66-NH_2_ was characterized using SEM, EDS, and SWASV. The SEM image revealed that there was no change in the octahedral-like morphology of UiO-66-NH_2_ after Pb^2+^ adsorption (Fig. 5c), suggesting that the physical structure of the MOF remained intact during the adsorption process. In addition, EDS analysis of the Pb^2+^-adsorbed UiO-66-NH_2_ (MOF-Pb; Fig. 5d) exhibited peaks that were characteristic of O, Zr, Pb, C, and N. The weight% of Pb was measured at 6.81%, indicating successful Pb^2+^adsorption. SWASV was performed in acetate buffer (pH 4.5) to further confirm Pb^2+^ adsorption. No oxidative peaks were observed for the pure MOF; in contrast, the MOF-Pb exhibited a well-defined Pb^2+^ oxidation peak at a potential of −0.59 V (Fig. 5e). The results of the EDS and SWASV analyses indicate that Pb^2+^ was successfully adsorbed onto UiO-66-NH_2_.

The functionalization of CEA-specific aptamers onto UiO-66-NH_2_ was subsequently investigated using FT-IR, DLS, and zeta potential measurement. Figure 5f presents the FT-IR spectra of the synthesized MOF, MOF-Pb, and MOF-Pb-Apt. In the pristine MOF, characteristic peaks were observed at 1101 cm^−1^ (C-N stretching of the -NH_2_ groups on the aromatic ring), 1656 cm^−1^ (C = O stretching of the carboxyl groups in the linker), 3465 cm^−1^ (N-H stretching of the -NH_2_ groups), and 768 cm^−1^ (Zr-O vibrations). The weakening of C = O and N-H stretching at 1656 and 3465 cm^−1^ was observed after Pb^2+^ adsorption due to interaction between the metal ions and the amine and carboxyl groups of UiO-66-NH_2_. In addition, the FT-IR spectrum of MOF-Pb-Apt exhibited a broad absorption peak at 1059 cm^−1^ that corresponds to P-O stretching vibrations from the phosphate groups in the aptamer, indicating successful aptamer immobilization on the MOF-Pb composite^47^. DLS analysis (Fig. 5g) revealed the hydrodynamic diameters of the MOF, MOF-Pb, and MOF-Pb-Apt to be 217.5 ± 10.8 nm, 254.4 ± 15.1 nm, and 264.9 ± 9.0 nm, respectively. The increases in particle size following Pb^2+^ adsorption and subsequent aptamer functionalization once again confirm the successful modification of the MOF surface. Zeta potential measurements of MOF, MOF-Pb, and MOF-Pb-Apt are presented in Fig. 5h. The pristine MOF exhibited a positive zeta potential of + 51.3 ± 0.7 mV, which was attributed to the protonation of -NH_2_/-NH- groups^57^. Upon the adsorption of Pb^2+^, the zeta potential became negative (−23.1 ± 0.6 mV), indicating that the MOF-Pb had been successfully prepared. The negative zeta potential of MOF-Pb-Apt further increased to −38.5 ± 2.0 mV due to the negatively charged phosphate backbone of the aptamer^46,58^. These results provide further confirmation of the successful adsorption of Pb^2+^ onto UiO-66-NH_2_ as well as subsequent aptamer functionalization.

### Parameter optimization

Key fabrication parameters that can influence the optimal performance of the sensor for CEA detection include hydroxyl group activation, APTES concentration, nanoMIPs concentration, and rebinding time; these parameters were systematically optimized to determine the ideal steps for the preparation of nanoMIPs/APTES/OH/MWCNTs/SPCE. Electrochemical measurements were conducted using a 2.5 mM [Fe(CN)_6_]^3−/4−^ redox probe.

**Fig. 6:**
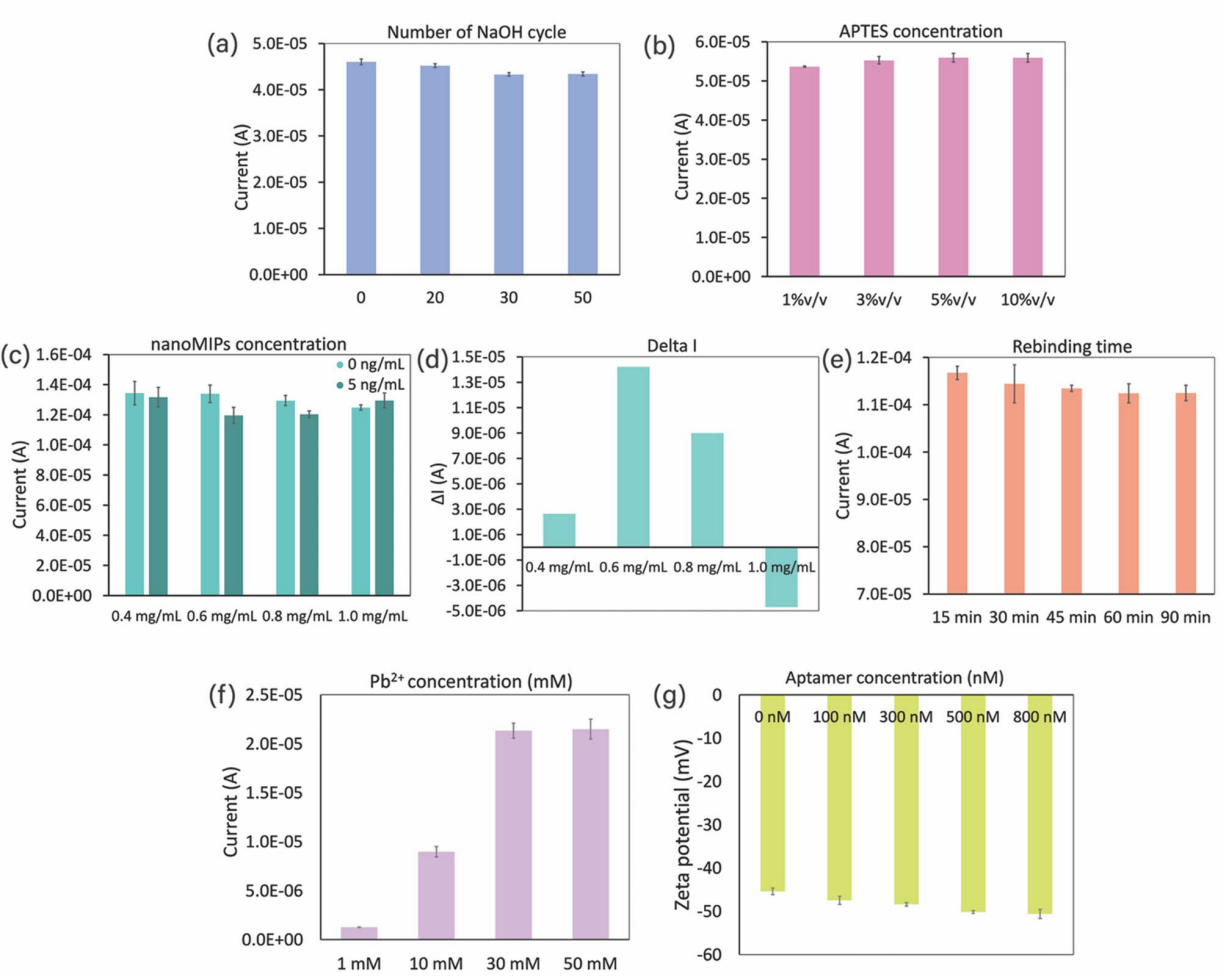
Effects of (**a**) the scan cycle number for hydroxyl group generation, (**b**) APTES concentration, (**c** and **d**) nanoMIPs concentration, (**e**) rebinding time, (**f**) lead ion concentration, and (**g**) aptamer concentration on sensor fabrication.

As hydroxyl group activation on the electrode surface was accomplished by using CV in NaOH solution, the number of CV scan cycles was optimized to maximize hydroxyl group generation. The number of scan cycles was varied across 0, 10, 30, and 50 cycles as demonstrated in Fig. 6a. There was a decreasing trend in current response as the number of CV scan cycles increased, highlighting the successful formation of hydroxyl groups on the electrode surface. However, the current response plateaued after 30 cycles, with no further significant decreases observed at 50 scan cycles. Consequently, 30 CV scan cycles in NaOH solution were determined to be the optimum for hydroxyl group activation.

The effect of APTES concentration on electrode modification was investigated using CV (Fig. 6b). Increasing current responses were observed as the APTES concentration increased to 3% v/v. However, further increases in APTES concentration to 10% v/v did not elicit any further significant enhancements in current response. The relatively stable current response beyond an APTES concentration of 3% v/v may be attributed to the limited surface area available for APTES functionalization on the electrode. Consequently, an APTES concentration of 3% v/v was selected as the optimal concentration for the preparation of APTES/OH/MWCNTs/SPCE.

The effect of nanoMIPs concentration on sensor performance was investigated by varying the concentration of nanoMIPs: 0.4, 0.6, 0.8, and 1.0 mg/mL of nanoMIPs were immobilized onto the APTES/OH/MWCNTs/SPCE. Each condition was tested with CEA concentrations of 0 ng/mL and 5 ng/mL; the corresponding SWV responses are presented in Fig. 6c. The results for the blank samples (CEA at 0 ng/mL) exhibited a decrease in current response with increasing nanoMIPs concentration due to the greater coverage of nanoMIPs on the electrode. These differences were normalized by evaluating the sensor performance in terms of the current change (ΔI) observed between the blank and CEA (5 ng/mL) samples (Fig. 6d). The highest ΔI was observed at a nanoMIPs concentration of 0.6 mg/mL, indicating that optimal sensitivity had been achieved. Higher concentrations of nanoMIPs did not promote CEA binding efficiency as indicated by the decrease in ΔI. Indeed, excessive nanoMIPs concentrations may result in loosely bound particles on the electrode surface, which could detach during the rebinding process, leading to a reduced or even negative ΔI. Therefore, 0.6 mg/mL nanoMIPs was chosen as the optimal concentration for sensor fabrication.

Finally, the rebinding time was varied (15, 30, 45, 60, and 90 min) to investigate its effect on sensor performance. Figure 6e shows that the SWV current responses decreased with increasing rebinding time, reflecting enhanced CEA binding to the nanoMIPs. However, saturation was observed at 60 min: extending the rebinding time to 90 min did not result in further significant improvements. Thus, a rebinding time of 60 min was chosen as the optimal condition for CEA detection.

For the preparation of the MOF-Pb-Apt signal probe, two critical parameters including lead ion concentration and aptamer concentration were optimized to enhance the sensor’s performance.

Since the amount of Pb^2+^ loaded affects the sensitivity of the signal response, the concentration of Pb^2+^ was optimized to achieve the best performance of the proposed sensor. UiO-66-NH_2_ was loaded with Pb^2+^ at concentrations of 1, 10, 30, and 50 mM. After removing the excess Pb^2+^, the synthesized MOF-Pb prepared at different Pb^2+^ concentrations was characterized using SWASV in acetate buffer (pH 4.5). Figure 6f compares the oxidative current responses of Pb^2+^ from MOF-Pb prepared at different loading concentrations. As expected, the current response increased with Pb^2+^ concentration from 1 to 30 mM. However, a further increase to 50 mM did not result in any significant change compared with 30 mM. This plateau in current response can be attributed to the limited number of active sites on UiO-66-NH_2_. Therefore, 30 mM Pb^2+^ was selected as the optimal concentration for signal probe preparation.

As the aptamer is the key element for the specific binding of the signal probe toward the target CEA, its concentration was optimized to improve sensor performance. The synthesized MOF-Pb was functionalized with the CEA-specific aptamer at concentrations of 0, 100, 300, 500, and 800 nM, and the corresponding zeta potential values were compared (Fig. 6g). A more negative zeta potential was observed as the aptamer concentration increased from 100 to 500 nM, indicating a higher amount of aptamer immobilized onto UiO-66-NH_2_. However, no significant change in zeta potential was observed when the concentration was further increased to 800 nM, which can be attributed to the limited surface area of UiO-66-NH_2_. Therefore, 500 nM was selected as the optimal aptamer concentration for immobilization.

### Performance of the developed nanoMIPs-aptamer sandwich assay for CEA detection

**Fig. 7:**
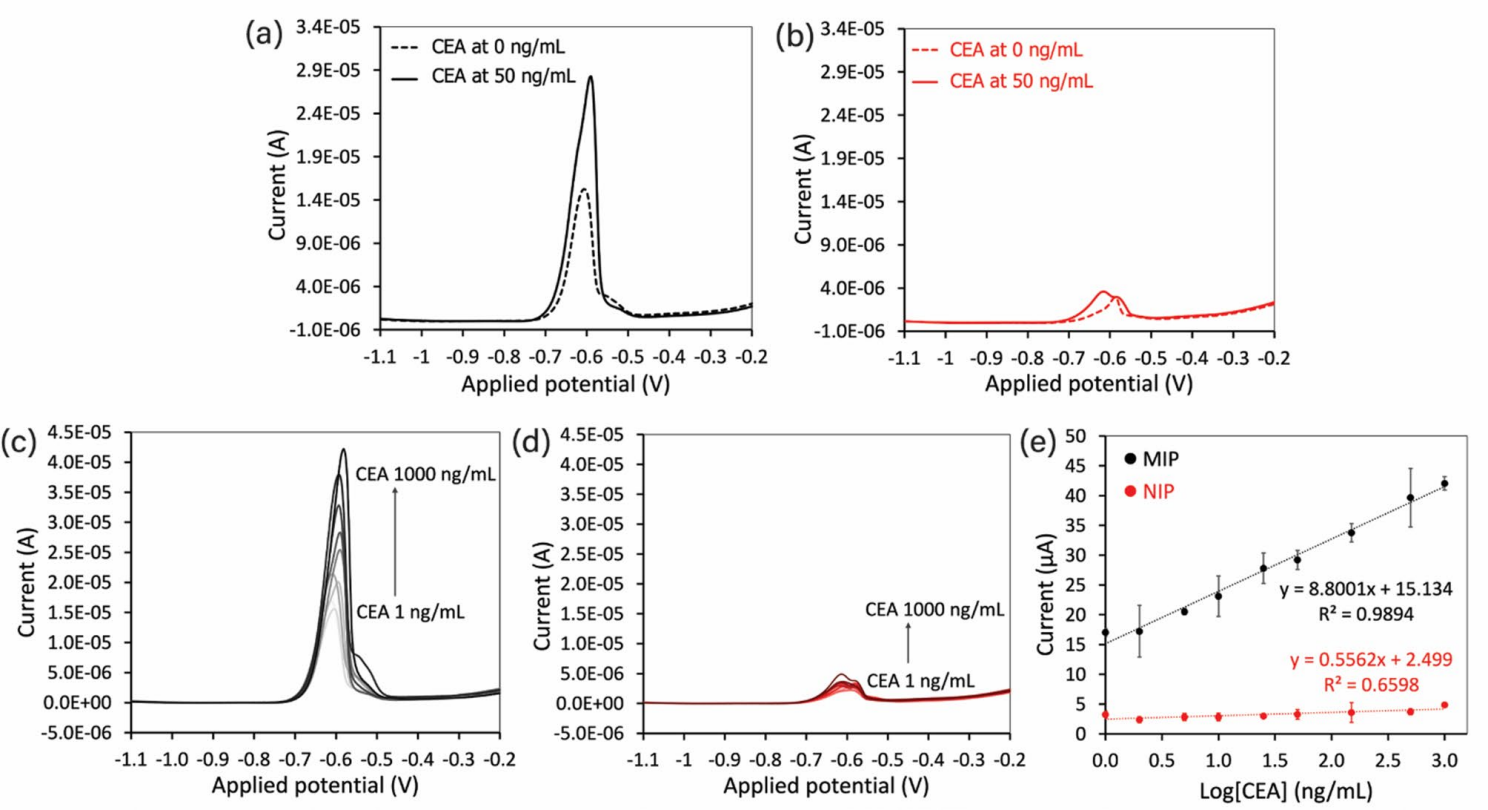
Comparison of voltammograms obtained from the (**a**) nanoMIPs-aptamer and (**b**) nanoNIPs-aptamer sandwich assays after testing with CEA at concentrations of 0 and 50 ng/mL. SWASV responses of the proposed (**c**) MIP sensor and (**d**) NIP sensor tested across a variety of CEA concentrations. (**e**) The corresponding standard curves of the current responses against the CEA concentrations (log-scale).

Following the successful preparation of the nanoMIPs/APTES/OH/MWCNTs/SPCE and the signal probe (MOF-Pb-Apt), both components were subsequently used in a nanoMIPs-aptamer sandwich assay for CEA detection. The platform was tested using CEA concentrations of 0 and 50 ng/mL; the results were compared to a control system using a nanoNIPs-aptamer sandwich assay. SWASV was performed to measure the oxidative current response of Pb^2+^, which corresponds to the amount of CEA bound to the electrode surface. Figure 7a presents the results obtained from the nanoMIPs-based platform. Oxidative current responses of Pb^2+^ were detected after exposure to both 0 and 50 ng/mL CEA. The response at 0 ng/mL CEA was considered to be the background response; this represents the residual template CEA molecules that remained following the polymerization process. There was a clear increase in current response in the sample with 50 ng/mL CEA, suggesting that CEA had been successfully bound to the imprinted sites, facilitating the formation of the sandwich complex. In contrast, the control experiment using the nanoNIPs-based platform (Fig. 7b) exhibited minimal current responses at both concentrations. Although a slight increase in the current response of the 50 ng/mL CEA sample was observed, this was likely due to non-specific adsorption. These findings demonstrate the efficiency of the nanoMIPs-aptamer sandwich assay for CEA detection.

### Analysis of the performance of the sensor for CEA detection

The performance of the developed sensor for CEA detection was systematically evaluated under optimized conditions. A range of CEA concentrations from 1 to 1000 ng/mL was tested; each was followed by the addition of MOF-Pb-Apt to form the sandwich complex. SWASV measurements were performed in acetate buffer (pH 4.5). The resulting voltammograms corresponding to each CEA concentration are shown in Fig. 7c and d for the nanoMIPs-based and nanoNIPs-based platforms, respectively. The corresponding calibration curves between the current responses and the CEA concentrations (log-scale) are presented in Fig. 7e. The oxidative current response of the Pb^2+^ ions obtained from the nanoMIPs-based platform increased significantly with increasing CEA concentration, suggesting that the signal originated from the specific binding of CEA to the nanoMIPs-based recognition sites. The developed sensor exhibited a linear detection range from 1 to 1000 ng/mL, represented by a linear trend that can be described by the regression equation I (µA) = 8.8001 × log[CEA] + 15.134 (R^2^ = 0.9894). In contrast, only a slight increase in the current response of Pb^2+^ ions was observed for the nanoNIPs-based platform. The corresponding linear regression equation was found to be I (µA) = 0.5562 × log[CEA] + 2.499 with a correlation coefficient of R^2^ = 0.6598. Statistical analysis revealed that the significance of the correlation had a p-value of 0.00795. The sensitivity of the nanoMIPs-based platform was found to be approximately 15.8 times higher than that of the nanoNIPs-based platform, clearly demonstrating the efficient and selective binding of the nanoMIPs sensor toward CEA. The limit of detection (LOD) was determined to be 1.4 ng/mL, calculated using the formula 3σ/slope, where σ represents the standard deviation of the blank measurements. The higher LOD compared to the lowest experimentally tested concentration within the linear range can be attributed to baseline variation and the moderate slope. However, this LOD is lower than the cut-off value of 5 ng/mL for clinical CEA diagnostics, highlighting the potential applicability of the proposed sensor for CEA detection.

The linear detection range and LOD obtained in this study were comparable to or better than those reported for previously published electrochemical MIP-based sensors for CEA detection (Table 1). Although the LOD reported in this study is not the lowest among previous works, the obtained linear detection range covers the clinically relevant concentration range (5 ng/mL to 1000 ng/mL), typically observed in cancer patients^16^. This wide detection range eliminates the need for tedious sample dilution steps. In contrast, most previously reported electrochemical MIP-based sensors for CEA detection primarily relied on label-free strategies. These studies focused on enhancing sensor sensitivity by modifying electrode surfaces with various nanomaterials and incorporating MIPs to provide specific recognition sites for CEA. However, their detection principle was largely based on the inhibition of electron transfer of redox compounds, such as Ru (III)^23^, iodine^24^, and the commonly used ([Fe(CN)_6_]^3−/4−^) probe^25–27^. Apart from using redox probes in the electrolyte, cobalt-based metal-organic frameworks (Co MOFs) have also been reported as modifier on SPCEs to serve as detection signals^28^. Although electrochemical label-free detection is convenient and widely used, nonspecific interferences in complex biological samples have been reported^23,24,29^, leading to reduced analytical performance. In contrast, the nanoMIPs-aptamer sandwich assay presented in this work enables the incorporation of a signal probe into the detection system. The use of a signal probe not only enhances the sensor’s sensitivity but also provide more selective recognition of CEA. In addition, most previously reported electrochemical MIP-based sensors for CEA detection employed electropolymerization to produce thin-film MIP on the electrode surface^23–29^. Although this technique is suitable for protein imprinting and can be applied to a variety of electroactive monomers, it requires polymerization on each individual electrode. This not only results in poor reproducibility but also makes the process time-consuming and challenging to scale up for large-scale production. In addition to thin film MIPs, core-shell molecular imprinting has also been proposed to improve the sensitivity of CEA detection^32^. Although this technique addresses the challenge of mass production, the signal measurement still relies on the inhibition of electron transfer of redox compound, [Fe(CN)_6_]^3−/4−^. In contrast, the present work employs nanoMIPs synthesized via solid-phase synthesis, which addresses the challenges associated with mass production. The CEA-specific nanoMIPs can be synthesized on a large scale for sensor fabrication, thereby overcoming the problem of poor reproducibility. Moreover, using nanoMIPs instead of thin-film MIP avoids the time-consuming process of large-scale sensor fabrication, as nanoMIPs can be immobilized on multiple electrodes simultaneously. In addition, since nanoMIPs are in nanoparticle form, unlike rigid MIP films on electrode surfaces, they can be applied to a variety of transducers, enabling broader applications. Furthermore, the sandwich format allows for the integration of signal probe, making this platform adaptable for multiplex biomarker detection, which remains a significant challenge for conventional MIP thin-film sensors.

**Table 1:** Comparison of the CEA detection performance of several electrochemical MIP-based sensors.

Electrode modification	Monomer	Electro-chemical method	Linear range (ng/mL)	LOD (ng/mL)	Ref.
Ag-SPE/–/PPy	Pyrrole	CV, EIS, SWV	0.00005–0.00125	-	Moreira et al.^23^
pFTO/hCCI/CEA-APBA/PAP	Amino phenol	EIS	2.5–1500	3	Truta et al.^24^
MIP/Au-SPEs	Gallic acid	EIS	1–1000	1	Carneiro et al.^25^
MIP-AuNPs/PTh/GCE	Dopamine	DPV	0.001–1000	0.000259	Lai et al.^26^
Bio-MIP-ePADs	Dopamine	DPV	1–500	0.32	Qi et al.^27^
MIP/CS/Co MOF-IL/SPCE	O-PD	DPV	0.0001–10	0.000024	Luo et al.^28^
PoPD MIP/Ti_3_C_2_T_x_ MXene/Silver strip	O-PD	EIS	10–100	9.41	Hadian et al.^29^
SiO_2_@AgNPs@MIP-CEA/SPGE SiO_2_@AgNPs@MIP-CRP/SPGE	IDA/EGDMA	EIS	0.0001–10	CRP = 0.000017 CEA = 0.000019	Somnet et al.^32^
nanoMIPs/APTES/OH/MWCNTs/SPCE and MOF-Pb-Apt CEA	nanoMIPs (AA, BIS, NIPAm, TBAm)	SWASV	1–1000	1.4	This work

*Ag-SPE* silver screen-printed electrodes, *PPy* polypyrrole, *EIS* electrochemical impedance spectroscopy, *pFTO/hCCI* pretreated fluorine doped tin oxide-glass modified with a homemade carbon ink, *APBA* 3-aminophenylboronic acid monohydrate, *PAP* polyaminophenol, *Au-SPEs* gold screen-printed electrodes, *AuNPs* gold nanoparticles, *PTh* polythionine, *DPV* differential pulse voltammetry, *Bio-MIP-ePADs* MIPs on movable valve microfluidic paper-based electrochemical device, *O-PD* O-phenylenediamine, *CS* chitosan, *Co MOF-IL* cobalt metal-organic framework-ionic liquid nanocomposite, SiO2@AgNPs Silica dioxide coated with silver nanoparticles.

### Selectivity testing

**Fig. 8:**
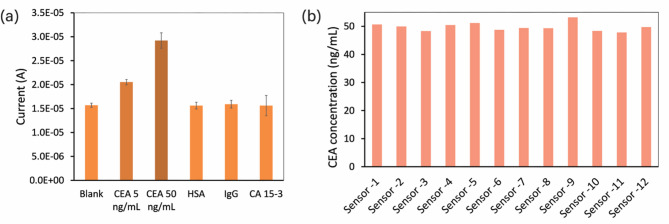
(**a**) Response of the prepared sensor to the blank sample (CEA at 0 ng/mL), CEA (5 ng/mL), CEA (50 ng/mL), HSA (0.1 mg/mL), IgG (0.1 mg/mL), and CA 15 − 3 (30 U/mL). (**b**) Comparison of the responses from 12 independently fabricated sensors after testing on 50 ng/mL CEA samples.

The selectivity of the proposed sensor was investigated by comparing its response toward CEA as well as other potential interferences. The nanoMIPs-aptamer sandwich assay was performed on a blank sample, 5 and 50 ng/mL CEA samples, human serum albumin (HSA, 0.1 mg/mL), immunoglobulin G (IgG, 0.1 mg/mL), and cancer antigen 15 − 3 (CA 15 − 3, 30 U/mL). Their respective oxidative current responses are presented in Fig. 8a. There was a noticeable increase in the oxidative current response of the CEA samples compared to the blank; in contrast, no significant changes were observed in the presence of any other interfering substances. These findings highlight the high selectivity of the proposed sensor toward CEA.

### Reproducibility testing

Reproducibility is a critical component of sensor development. The reproducibility of the proposed nanoMIPs-aptamer sandwich assay was evaluated using 12 independently fabricated sensors. Each sensor was tested on a 50 ng/mL CEA sample. Figure 8b shows that all sensors exhibited highly consistent responses with a low relative standard deviation (RSD) of 2.97%. This finding highlights the high reliability and consistent response of sensors fabricated using the methods proposed in this work.

### Application to serum samples

The nanoMIPs-aptamer sandwich assay was applied to the detection of CEA in human serum samples. Commercial human serum was spiked with CEA at concentrations of 5, 10, and 25 ng/mL. Although the proposed sensor demonstrated satisfactory selectivity toward CEA, it is important to note that the concentration of human serum albumin (HSA) in real human serum is considerably high (35–50 mg/mL). Such high levels of HSA may interfere with the biosensor response through nonspecific binding or surface blocking. To address this issue, a pretreatment step using a molecular weight cut-off (MWCO) filter was applied to reduce the HSA concentration in the serum samples. After pretreatment to minimize the effects of HSA, the samples were analyzed using the prepared sensor to evaluate its performance in clinical applications. The RSD values of the responses ranged from 3.68 to 7.63%, and the average recoveries obtained were between 98.12 and 103.24%. Statistical t-tests revealed no significant differences between the spiked concentrations and the measured values at a 95% confidence level (p-value ≥ 0.05; Table 2). These results emphasize the potential application of the developed sensor to the accurate and precise detection of CEA in human serum samples.

**Table 2:** Application of the proposed nanoMIPs-aptamer sandwich assay to CEA detection in human serum samples.

Sample	Added (ng/mL)	Found (ng/mL)	%RSD	%Recovery	*P*-value
1	5.00	5.12		102.42	
2	5.00	5.57		111.50	
3	5.00	4.79		95.81	
Average	5.00	5.16	7.63	103.24	0.5524
4	10.00	10.01		100.11	
5	10.00	9.61		96.08	
6	10.00	10.34		103.42	
Average	10.00	9.99	3.68	99.87	0.9554
7	25.00	24.42		97.66	
8	25.00	25.74		102.96	
9	25.00	23.43		93.74	
Average	25.00	24.53	4.72	98.12	0.5552

*Statistical one-sample t-test at 95% confident level.

## Conclusions

This study presents the development of a nanoMIPs-aptamer sandwich assay for the quantitative detection of the clinically significant biomarker CEA using nanoMIPs as a synthetic recognition element on the electrode surface and MOF-Pb-Apt as an electrochemical signal probe. The electrochemical measurements demonstrated the excellent performance of the developed sensor for CEA detection. The linear detection range of the sensor covers most clinically relevant concentrations and exhibits a LOD lower than the diagnostic cut-off value for CEA. Furthermore, the proposed sensor exhibited high sensitivity, reproducibility, and reliability when tested on real samples, highlighting its potential applications in clinical diagnostics. Using molecularly imprinted artificial receptors eliminates the issues associated with traditional antibodies, such as high costs and the need for professional operation, allowing for the fabrication of low-cost and easy-to-use sensors. Nevertheless, some limitations of the proposed sensor should be acknowledged. Direct validation against a clinical reference method such as ELISA was not performed due to resource and regulatory constraints. In addition, although pretreatment with a MWCO filter successfully reduced interference from abundant serum proteins such as HSA, the possibility of nonspecific effects at very high protein concentrations cannot be completely excluded. Looking forward, future research should include direct comparisons with ELISA or other gold-standard methods to further strengthen the impact of the sensor. Moreover, the use of molecular modeling could help identify suitable monomers and optimize the recipe for nanoMIP polymerization, thereby minimizing nonspecific binding and further enhancing sensor performance. Additionally, the proposed sandwich assay can also be further developed for the simultaneous detection of multiple biomarkers through the use of different metal ions (e.g., Cd^2+^, Cu^2+^, and Zn^2+^) as distinguishable signal reporters. This study demonstrates the excellent potential of the proposed nanoMIPs–aptamer sandwich assay for use in commercial electrochemical devices for cancer monitoring and early diagnosis.

## Data Availability

All relevant data are contained within the article.
